# Recurrence of Glomerular Diseases (GN) After Kidney Transplantation: A Narrative Review

**DOI:** 10.3390/jcm14186686

**Published:** 2025-09-22

**Authors:** Abbal Koirala, Aditi Singh, Duvuru Geetha

**Affiliations:** Division of Nephrology, Johns Hopkins School of Medicine, 5501 Hopkins Bayview Circle, Floor 1, Baltimore, MD 21224, USA; akoiral1@jh.edu (A.K.); asing148@jh.edu (A.S.)

**Keywords:** glomerular disease (GN), IgA nephropathy (IgAN), focal segmental glomerulosclerosis (FSGS), membranoproliferative glomerulonephritis (MPGN), C3 glomerulopathy (C3G), membranous nephropathy (MN), recurrent lupus nephritis (RLN), anti-glomerular basement membrane disease (anti-GBM disease), ANCA-associated vasculitis (AAV)

## Abstract

Recurrence of the original glomerular disease (GN) poses a significant threat to kidney transplant function and longevity. The probability and severity of this recurrence vary, with C3 glomerulopathy and certain forms of FSGS exhibiting particularly high rates. Kidney transplant GN recurrence risk hinges on the characteristics of the initial GN, recipient/donor genetics, recipient age, donor type, end-stage kidney disease (ESRD) progression rate, and proteinuria levels. Standard immunosuppression has limited efficacy in preventing primary disease recurrence; however, agent selection and induction therapy can influence the risk for specific GNs. Diagnosing recurrent GN involves a comprehensive approach, including clinical evaluation, laboratory tests (such as proteinuria, hematuria, and specific biomarkers like anti-PLA2R for membranous nephropathy or complement for C3G), and, critically, an allograft biopsy analyzed with light, immunofluorescence, and electron microscopy. Treatment strategies are evolving towards targeted therapies, such as rituximab for antibody-mediated GN and complement inhibitors for C3G, moving away from broad immunosuppression. This narrative literature review provides practical monitoring algorithms for post-transplant settings, synthesizing information on the incidence, predictors, diagnostic strategies, and therapeutic options for various glomerular disease subtypes. The methodology involved searching MEDLINE, Embase, and Cochrane databases from 1996 to 2025, prioritizing systematic reviews, cohort studies, registries, and interventional reports. Eligibility criteria included adult transplant recipients and English-language reports on recurrent glomerular disease outcomes, excluding most single-patient case reports. Limitations include potential selection bias, omission of relevant studies, and the absence of a formal risk-of-bias assessment or meta-analysis. The evidence base is heterogeneous, with inconsistent outcome reporting and scarce randomized controlled trials. Future efforts should focus on developing predictive biomarkers, standardizing diagnostic and response criteria, conducting multicenter prospective cohorts and pragmatic trials, and creating shared registries with harmonized data.

## 1. Introduction

Glomerular diseases (GN) are a leading global cause of end-stage renal disease (ESRD). In the United States, GN accounts for 7% of new dialysis patients and 13% of kidney transplant recipients, underscoring its substantial global impact on chronic kidney disease [1].

Despite the considerable benefits of kidney transplantation, the long-term survival of the transplanted allograft is seriously and continuously threatened by the recurrence of the original GN or the development of de novo GN. This recurrence is a key contributor to early allograft failure, ranking as the third leading cause of graft loss within ten years after renal transplantation [1,2,3,4,5]. GN recurrence post-transplant varies from 3 to 15% generally [1], but certain types, like dense deposit disease, can reach 100% [2]. Common GNs (IgA nephropathy, focal and segmental glomerulosclerosis, membranous nephropathy) also show notable recurrence, although usually lower than 100%. This variability underscores the importance of early detection and management to improve long-term outcomes for kidney transplant recipients with GN.

The timing of recurrence varies. While risk increases with time, some GNs, such as focal and segmental glomerulosclerosis (FSGS) and C3 glomerulopathy (C3G), can recur within days or weeks. This bimodal pattern suggests different mechanisms: early recurrence due to active, persistent systemic factors (e.g., permeability factors, antinephrin antibodies in FSGS, complement dysregulation in C3G); later recurrence due to gradual reestablishment of the original disease process, possibly influenced by the allograft environment or long-term interactions with the immunosuppressive regimen.

The recognition of recurrent GN as a major risk to long-term graft survival represents a shift in post-transplant care. While preventing and treating acute rejection remains vital, managing disease recurrence is now equally crucial for durable allograft function in patients transplanted for GN. This necessitates a deeper understanding of recurrence factors for each specific GN and the development of targeted preventive and therapeutic strategies.

A fundamental challenge is the ongoing presence of the underlying systemic disease, which is not cured by transplantation. The transplanted kidney is susceptible to the same disease processes that damaged the original kidneys. This vulnerability requires rigorous patient care, including a thorough pre-transplant risk assessment to identify individuals at higher recurrence risk, as well as aggressive, individualized post-transplant monitoring and management. Understanding that transplantation addresses organ failure but not necessarily the systemic disease is vital for optimizing long-term outcomes for transplant recipients.

## 2. Methods

This narrative literature review aims to provide practical monitoring algorithms for the post-transplant setting. It synthesizes information regarding the incidence, predictors, diagnostic strategies, and therapeutic options for various glomerular disease subtypes. The review’s methodology involved searching MEDLINE, Embase, and Cochrane databases for publications from 1996 to 2025. The search utilized controlled vocabulary and keywords related to glomerulonephritis, recurrence, and kidney transplantation. Priority was given to systematic reviews, cohort studies, registries, and interventional reports, with findings narratively synthesized by disease entity, emphasizing clinical applicability. Eligibility criteria included adult transplant recipients and reports containing data on the incidence, predictors, diagnosis, treatment, or outcomes of recurrent glomerular disease. Only English-language reports were included, and single-patient case reports were excluded unless uniquely informative. However, this review has several limitations, including potential selection bias, possible omission of relevant studies, and the absence of a formal risk-of-bias assessment or meta-analysis. The evidence base is heterogeneous, with variability in diagnostic criteria and outcome definitions. Most data are derived from retrospective cohorts and transplant registries, which are subject to indication and survivorship biases. Outcome reporting is inconsistent, and response criteria are not standardized. Randomized controlled trials in kidney transplant recipients are scarce, and generalizability from native kidney disease studies is uncertain. The strength of evidence was qualitatively graded as high, moderate, or low, considering study design, sample size, consistency, and directness.

Future initiatives should prioritize the development and validation of predictive biomarkers for recurrence risk stratification. Additionally, efforts should focus on establishing standardized diagnostic and response criteria specifically adapted for the transplant setting. Conducting multicenter prospective cohort studies and pragmatic trials, alongside the creation of shared registries with harmonized data elements, are also crucial next steps.

### 2.1. General Epidemiology and Impact of Recurrent GN

#### 2.1.1. Overall Prevalence of GN Recurrence Post-Transplant

The recurrence of GN after kidney transplantation is observed in 3% to 15% of cases. However, this prevalence is probably underestimated for several reasons. Selection bias in patient listing for transplantation is a major factor, as systematic variations in the selection and listing of ESRD patients with different GN subtypes and observation period can distort reported incidences.

Another critical factor is the inconsistency in biopsy practices across transplant centers. Centers that regularly perform “protocol” biopsies (scheduled biopsies regardless of symptoms) report higher recurrence rates than those that only perform “for-cause” biopsies (biopsies performed due to clinical signs of dysfunction). This suggests that many recurrences may be subclinical and only identifiable through routine histological examination. Furthermore, the diagnosis of recurrent GN may require electron microscopy, which is not routinely performed. Additionally, difficulties in accurately determining the primary GN diagnosis in the native kidney can also lead to underestimation.

Recurrent glomerular disease increases in prevalence over time, becoming the second leading histopathological diagnosis after antibody-mediated rejection at 10 years post-transplant. The risk of recurrence is generally proportional to time since transplantation, with most allograft failures occurring within 3–5 years of recurrence. However, C3G and FSGS can recur earlier, sometimes within months. This temporal variability highlights the need for tailored monitoring strategies tailored to the specific GN subtype.

#### 2.1.2. Impact of Recurrence on Allograft Survival and Patient Outcomes

Recurrent GN is a substantial contributor to premature allograft failure, representing a critical challenge in the long-term success of kidney transplantation. Patients who experience recurrent GN are twice as likely to lose their allograft compared to those who do not, with approximately 45% of affected grafts failing within 5 years of recurrence [3,6,7,8]. This profound impact on graft longevity positions recurrent disease as the third most common cause of graft failure 10 years after renal transplantation, following death with a functioning graft and chronic rejection.

The severity of the impact of recurrence on graft survival varies considerably depending on the specific GN subtype. For instance, IgA Nephropathy (IgAN) is generally associated with better long-term allograft and patient outcomes compared to other GN subtypes, even after recurrence. In contrast, MPGN (membranoproliferative glomerulonephritis), particularly dense deposit disease (DDD), is associated with a substantially poorer allograft survival rate following recurrence. FSGS also presents a high risk of allograft failure upon recurrence, with rates as high as 50%. This differential impact highlights that while recurrence is a broad concern, its specific consequences are highly dependent on the underlying disease pathology.

The clinical implications of these observations are significant. The high rate of graft loss associated with recurrence means that patients with certain GN types face an ongoing battle even after receiving a new kidney. This situation creates a complex dynamic where the initial success of transplantation must be continuously protected against the re-emergence of the original disease. The variability in outcomes across different GN subtypes suggests that a “one-size-fits-all” approach to post-transplant management is insufficient. Instead, management strategies must be precisely adapted to each GN type’s specific characteristics and recurrence risks. This tailored approach is crucial for maintaining graft function and enhancing the overall quality of life and long-term survival for transplant recipients.

Key points are as follows:

- Recurrence of glomerular diseases (GN) after kidney transplantation is a significant factor in long-term graft loss, with varying rates depending on the specific GN subtype.
- Diagnosis relies on clinical suspicion (proteinuria, hematuria, declining graft function) and allograft biopsy, with protocol biopsies revealing more subclinical cases than “for-cause” biopsies.
- Management and monitoring strategies for recurrent GN are highly individualized based on the specific disease subtype, requiring a comprehensive understanding of each GN’s immunopathogenesis and risk factors.

### 2.2. Specific GN Subtypes and Their Recurrence Characteristics (See Figure 1 and Table 1, Table 2, Table 3 and Table 4)

#### 2.2.1. IgA Nephropathy (IgAN)

IgA nephropathy (IgAN) is the most common primary GN. It leads to ESRD in 15–20% of patients within 10 years, and 20–40% within 20 years of diagnosis.

Prevalence and Time to Recurrence: IgAN recurrence after kidney transplant is common, with histological recurrence rates of up to 51% at 5 years. Clinical recurrence often appears after 5 years. Rates vary from 10% to 30% in “for-cause” biopsy studies and from 25% to 53% in “protocol” biopsy studies, indicating that many subclinical cases are detected only through systematic histological examination [9,10,11,12]. The median recurrence time is approximately 59 months (range, 16–90 months).

Graft Outcome: Recurrent IgAN is common, negatively impacting graft survival and leading to allograft loss in up to 40% of patients (60% due to recurrence). This significantly lowers 10-year graft survival, with a 5-year allograft failure rate of 42%. While recurrent IgAN is common, its long-term impact on allograft and patient outcomes is generally considered better compared to other GN subtypes [5,9,10,13,14].

Risk Factors:

- Recipient Factors: Younger age at IgAN diagnosis and at transplant [9,15], faster progression from IgAN to ESRD [15,16], pre-transplant tonsillectomy, prior kidney transplantation (43% increased risk), hemodialysis as KRT [15], and preemptive transplantation [9].
- Immunosuppression and Induction Therapy: Higher recurrence rates are associated with steroid-avoidance/early steroid withdrawal and no induction therapy. ATG induction, mycophenolate mofetil, anti-IL-2R antibody induction, and pre-transplant tonsillectomy may decrease recurrence. Post-transplant mTOR inhibitors increase recurrence risk [15,17].
- Immunological Factors: Pre- and post-transplant donor-specific antibodies (DSAs) significantly increase recurrence risk (HR 2.74 and 6.65, respectively [9]). Other risk factors include the presence of Gd-IgA1, IgG anti-Gd-IgA1, glycan-specific IgG antibodies, and soluble CD89 [18], as well as sIgA levels post-transplantation [19].
- Genetic Factors: IgAN is a genetically diverse polygenic disease involving MHC (HLA) and non-MHC susceptibility alleles. Lower HLA/HLA-DR mismatches and living donors are associated with higher recurrence rates [15].
- Clinical Course: Crescentic IgAN, an aggressive pre-transplant condition, increases the risk of early, aggressive recurrence. Recurrence is associated with higher proteinuria in the initial year post-transplant and a more rapid eGFR decline [20].

Histology: Histologically, recurrent IgAN is defined by dominant or co-dominant IgA mesangial staining on immunofluorescence, often accompanied by mesangial expansion and hypercellularity on light microscopy.

Immunological and Pathological Mechanisms: IgAN recurrence is a complex process involving the continued production and deposition of abnormal IgA1 (Gd-IgA1) and immune complexes in the mesangium of the transplanted kidney. IgG anti-Gd-IgA1 and glycan-specific IgG antibodies contribute to this. The “multi-hit hypothesis” highlights genetic and environmental interplay in the pathogenesis. Post-transplant DSAs increase risk, suggesting an alloimmune component that exacerbates the autoimmune process. Histologically, recurrent IgAN shows IgA deposition, mesangial hypercellularity, and segmental glomerulosclerosis in allograft biopsies [21,22].

##### Treatment of Recurrent IgAN Post-Transplantation

No standardized treatment exists for recurrent IgA nephropathy (IgAN) following kidney transplantation. Management primarily involves supportive care, with renin–angiotensin system (RAS) inhibitors (ACE inhibitors or ARBs) recommended to reduce proteinuria and protect kidney function [10].

For active or severe recurrence, a regimen of intravenous methylprednisolone (500 mg daily for three days at months 1, 3, and 5) combined with oral prednisone (0.5 mg/kg every other day for six months) may be considered [23]. However, more extensive randomized controlled studies are needed to establish a gold standard treatment.

In severe or treatment-resistant cases, particularly those with endocapillary proliferation or crescentic lesions, additional therapies like cyclophosphamide [24,25,26], rituximab [27,28], and, rarely, complement inhibitors such as eculizumab [29], have shown potential in limited studies and individual reports.

Tonsillectomy one year post-transplant has been linked to reduced histological IgAN recurrence (11.1% vs. 55.6%), decreased serum Gd-IgA1 levels, and reduced mesangial IgA and Gd-IgA1 immunoreactivity compared to controls [30]. Modifications to maintenance immunosuppression regimens, such as using everolimus with tacrolimus and corticosteroids versus MMF-based regimens with tacrolimus and corticosteroids, have shown a correlation with reduced recurrence in specific scenarios; however, further research is required to confirm their efficacy [31]. Dual BAFF-APRIL inhibitors [32,33], including Atacicept and Povitacicept, which are currently being investigated for treating native IgAN to reduce Gd-IgA1 immune complex production, may prove beneficial in preventing IgAN recurrence after kidney transplantation.

Screening and monitoring: Microhematuria and proteinuria screening: monthly for the first month, quarterly for the first year, then annually. Emerging biomarkers (IgA, IgA-IgG complexes, Gd-IgA1, anti-Gd-IgA1 autoantibodies) are investigational. New or worsening hematuria, proteinuria, or graft dysfunction necessitates allograft biopsy to confirm recurrence and exclude other causes [10,16].

#### 2.2.2. Focal Segmental Glomerulosclerosis (FSGS)

FSGS is the most frequent glomerular disease causing ESRD globally, posing a challenge in kidney transplantation due to high recurrence. It is a non-specific histological finding of various podocytopathies, and differentiating types is crucial for post-transplant care, as primary FSGS often recurs, unlike other forms [34,35].

Prevalence and Time to Recurrence: Recurrent FSGS (rFSGS) is a severe complication following kidney transplantation, with reported incidence rates ranging from 17% to 55% [36]. The recurrence rate in first kidney grafts for primary FSGS is estimated at 40–60%, and this can rise to over 80% in patients whose initial graft failed due to recurrent FSGS [37]. Typically, recurrence manifests early in the post-transplant period, with a median onset of 1.5 months. It can be aggressive, leading to nephrotic-range proteinuria within hours to days after transplantation. Most recurrences are observed within the first two years following kidney transplantation [36,37].

Graft Outcome: rFSGS frequently causes premature allograft failure, with a 5-year graft survival rate of 52% compared to 83% in non-recurrent cases [36]. Half of the patients experiencing recurrence lose their graft within 5 years, with most graft losses occurring within the first 2 years after disease recurrence. De novo FSGS shows slower progression and better long-term survival (60% vs. 33.3% probability of graft survival) than recurrent FSGS [38].

Risk Factors:

- Recipient Factors: rFSGS is highly influenced by prior allograft recurrence (80% risk). Other factors include younger age at onset, rapid ESRD progression, BMI at transplantation, and nephrotic syndrome with low serum albumin (<2.5 g/L) at diagnosis [39,40]. Pre-transplant antinephrin antibodies predict FSGS recurrence, showing high specificity [41,42]. In recurrence cases, allograft biopsies reveal co-localized glomerular deposition of nephrin and IgG, suggesting a pathogenic role needing further investigation [41].
- Genetic Factors: Familial FSGS, due to podocyte slit diaphragm protein mutations (excluding podocin), generally has low or no recurrence risk [43,44]. Pre-transplant genetic testing is crucial for risk assessment, identifying suitable living donors, and avoiding unnecessary immunosuppression.
- Histological Features: The native kidney’s FSGS subtype (e.g., collapsing, tip lesions) does not significantly impact recurrence risk or type of FSGS seen in the allograft [45].
- Donor Factors: rFSGS and donor type are debated. Some studies weakly link living donors to higher recurrence [40] while others find no independent association. Despite this, living donor kidney transplantation generally results in improved graft survival.

Immunological and Pathological Mechanisms: rFSGS is linked to circulating permeability factors like Soluble Urokinase-Type Plasminogen Activator Receptor (suPAR), Cardiotrophin-like Cytokine Factor 1 (CLCF-1), plasminogen activator inhibitor type-1 (PAI-1), and angiotensin II type 1 receptors (AT1Rs) [46]. This is supported by observations of recurrence despite immunosuppression, maternal–child transmission, and the effectiveness of plasmapheresis in reducing proteinuria and preventing recurrence [47]. Experimental evidence shows that FSGS patient serum induces proteinuria in rats [48,49] and affects podocytes in cell cultures [50].

Antinephrin antibodies are implicated in primary podocytopathies, found in 44% of adults with minimal change disease (MCD) and 9% with pFSGS in one study [51]. A link between antinephrin antibodies and disease activity was observed, hinting at a possible pathogenic function, though further investigation is needed. Another study identified them in 45% of MCD and 37.5% of pFSGS patients [52]. Furthermore, the presence of pre-transplant antinephrin antibodies has recently been linked to rFSGS.

Histology: The only initial finding in allograft biopsies for recurrent FSGS may be diffuse podocyte foot process effacement on electron microscopy, alongside new-onset progressive proteinuria.

##### Recurrence Prevention

Evidence regarding post-transplant recurrence prevention strategies is both limited and contradictory. KDIGO 2020 guidelines advise against routine pre-transplant plasma exchange (PLEX) or rituximab (RTX) to lower the risk of rFSGS (2D) [37,53]. Although previous studies found no significant effect of prophylactic PLEX on rFSGS, these studies were limited by small sample sizes, retrospective analyses, and lack of randomization [54]. Conversely, a recent retrospective study indicated that pre-transplant PLEX was associated with a reduced rFSGS (34.8% recurrence risk without PLEX vs. 9.4% with PLEX) [55]. Similarly, data on prophylactic RTX use are limited. A meta-analysis suggests that RTX, either alone or with PLEX, or PLEX alone, does not substantially decrease the risk of rFSGS [56].

##### Recurrence Management of Post-Transplant FSGS

Treatment options for rFSGS are limited and have not changed much. Daily proteinuria monitoring post-transplant is vital for early recurrence detection. Rituximab (RTX) and plasma exchange (PLEX) are common initial treatments for pFSGS recurrence (80% of cases). RTX acts on podocytes and depletes B cells, reducing permeability factors and antinephrin antibodies. PLEX removes circulating pathogenic factors. Early RTX response is attributed to its direct podocyte-stabilizing effect through SMPDL3b [57].

Immunoadsorption selectively removes immunoglobulins, preserving coagulation factors, unlike plasma exchange (PLEX), but is more expensive and less available, with no proven clinical superiority [58,59]. The TANGO study of 61 rFSGS patients treated with PLEX ± rituximab (RTX) showed 21% complete remission, 36% partial remission, and 43% no response [39]. A French study by Lanaret et al. [60] comparing standard care (PLEX, CNI, steroids) with or without RTX reported 46.6% complete and 33.1% partial remission rates, with significantly better 10-year graft survival in responders (64.7%) versus nonresponders (17.9%). For plasma exchange (PLEX) or rituximab (RTX)-unresponsive cases, alternative strategies, targeting specific mechanisms in podocytes are suggested, including ACTH gel [61,62], LDL pheresis [63,64,65], abatacept, and ofatumumab [66,67].

Screening and monitoring of rFSGS: For potential early, fulminant FSGS recurrence, high-risk patients need more frequent post-transplant proteinuria screening: daily for week 1, weekly for 4 weeks, then quarterly for a year, and annually thereafter. Close monitoring, allograft biopsy for confirmation, and prompt, intensive treatment are crucial [10,16]. Pre-transplant genetic testing is essential to rule out genetic causes of FSGS, which have a low likelihood of recurrence. Biomarkers like suPAR, CLC-1, AT1R-Ab, anti-CD40, IL-13, and antinephrin antibodies are being explored.

#### 2.2.3. Membranous Nephropathy (MN)

Membranous Nephropathy (MN), a major cause of kidney failure and nephrotic syndrome in adults, recurs post-transplant (10–45%, 31% cumulative incidence at 10 years). Recurrence averages 6.3 years, longer than other glomerular diseases. Early recurrence (6–12 months) implies the presence of pre-existing antibodies, while late onset (around 5 years) suggests new antibody production.

De novo MN, rare in transplanted kidneys, is PLA2R-negative and presents with proteinuria, leading to poorer outcomes than recurrent MN (which is PLA2R-positive). Often linked to alloimmune responses and anti-HLA antibodies, it may show concurrent antibody-mediated rejection histologically and lacks IgG4 dominance, differentiating it from primary MN. Its clinical course is more aggressive, with a higher risk of allograft loss [68,69].

Graft Outcome: While most studies on recurrent membranous nephropathy (MN) indicate no significant independent link with graft loss [70,71], de novo MN might carry a more severe prognosis for graft survival in certain situations. The reported graft failure rate for de novo MN is 11.7 per 100 person-years, markedly higher than the 3.7 per 100 person-years observed in recurrent MN [68].

Risk Factors:

- Immunological Factors: Elevated pre-transplant anti-PLA2R antibodies are the primary risk factor for recurrence, with a 70% risk in positive patients vs. 30% in negative [7,37,71,72,73]. High pre-transplant PLA2R levels and post-transplant persistence/reappearance indicate earlier and more aggressive disease.
- Recipient Factors: Higher MN post-transplant recurrence is linked to female sex, younger age [5], recipient HLA-A3 antigen [68], high pre-transplant proteinuria [72], and faster ESRD progression [71].
- Genetic Factors: Donor HLA-D and PLA2R1 risk alleles can increase MN recurrence [74,75].
- Donor Factors: Older studies suggest a higher recurrence rate in living- vs. deceased-donor transplants, especially living-related, implying a genetic link [68].

Immunological and Pathological Mechanisms: Recurrent membranous nephropathy (MN) is mainly caused by recipient anti-PLA2R IgG4 autoantibodies binding to donor kidney podocyte antigens like the M-type phospholipase A2 receptor (PLA2R). These antibodies, found in 70–80% of primary MN patients, are key to immune complex formation in recurrent MN, which then activate complement and lead to subepithelial immune deposit formation, podocyte injury, and proteinuria [76]. Anti-PLA2R antibody identification aids MN risk assessment, though new antigens still affect recurrence rates.

Histology: Recurrent MN is histologically characterized by subepithelial immune complex formation, granular IgG, and less frequent complement deposition along the glomerular basement membrane, and detectable PLA2R antigen in immune deposits. It may present subclinically, often detected on protocol biopsies before overt proteinuria [72,77].

##### Treatment of Post-Transplant Recurrent MN

Post-transplant recurrent membranous nephropathy is initially managed by optimizing conservative, antiproteinuric measures (ACE inhibitors/ARBs, strict adherence to the existing immunosuppressant regimen). Rituximab is the favored initial immunosuppressive therapy for proteinuria >1 g/day, showing better remission rates than conservative management [71,72,78]. Monitoring anti-PLA2R antibody levels post-transplantation is crucial for detecting recurrence, potentially requiring biopsy and more intensive treatment [78]. Regular antibody measurements are advised for PLA2R-associated disease for 6–12 months post-transplant. Biopsy should be considered if proteinuria is 0.3–1.0 g/day and antibody levels are rising or high [71,78].

NKF and KDIGO guidelines emphasize tailored treatment for refractory or relapsing disease, though rituximab is preferred when additional immunosuppression is required. Case reports and small series suggest obinutuzumab as a promising alternative to rituximab for post-transplant MN recurrence when rituximab is ineffective [79,80,81].

Screening and monitoring of recurrent MN: KDIGO guidelines suggest monthly proteinuria checks for 6–12 months post-transplant to screen for recurrent membranous nephropathy. For PLA2R disease, monitor anti-PLA2R antibodies monthly (high titers) to quarterly (undetectable) based on pre-transplant levels. Biopsy if proteinuria exceeds 1 g/day. Consider biopsy for rising/high anti-PLA2R titers even with proteinuria in the 0.3–1.0 g/day range, indicating early recurrence. For non-PLA2R MN, only proteinuria monitoring is recommended, with biopsy if it exceeds 1 g/day [78].

#### 2.2.4. Membranoproliferative Glomerulonephritis (MPGN)/C3 Glomerulopathy (C3G)

MPGN is classified as immune complex GN (ICGN) or complement-mediated GN, also known as C3 glomerulopathy (C3G), a rare, progressive kidney disease caused by dysregulation of the alternative complement pathway.

Prevalence and Time to Recurrence: MPGN recurrence post-transplant is 20–45%, higher in complement-mediated forms (C3G) and with monoclonal gammopathy (IC-MPGN). Polyclonal immune complex-mediated MPGN has a lower risk, while C3G recurrence can exceed 60–70%. DDD, a C3G subtype (formerly MPGN type II), has a near 100% late recurrence rate. C3G recurs early after transplant [37,82,83]. The median time to recurrence for C3G ranges from 1.1 to 28 months, with some recurrences as early as 9 days post-transplant [84].

Graft Outcome: MPGN recurrence, especially C3G, negatively impacts graft survival, which is poorer than other GN subtypes. Half of recurrent MPGN patients may lose allografts. C3G recurrence leads to graft failure in 11–77% (mostly >50%) [82,83,84,85,86]. Graft loss is more common and sooner in DDD than in C3 glomerulonephritis [84].

Risk Factors for IC-MPGN

- Recipient Factors: Lower BMI at transplant, crescents in original biopsy, more glomeruli with crescents in native biopsy [10,85], shorter dialysis duration before kidney transplantation [87], and prior graft loss from MPGN [10] are all factors.
- Immunological Factors: Lower serum complement and monoclonal gammopathy [1,53,82] correlate with higher recurrence and poorer outcomes. Recurrence is less common in polyclonal IC-MPGN, especially if the underlying cause is controlled [83].
- Donor Factors: Higher recurrence rates of ICGN have been linked to living-related allografts and preemptive transplantation [82].
  Risk Factors for C3G
- Recipient Factors: Young age at diagnosis, an aggressive course of native kidney disease, male sex, and pre-emptive transplantation are risk factors [53,84]. Some studies have noted low complements as risk factors at the time of transplantation [53]. Paraproteinemia-associated C3G can drive complement dysregulation and is associated with more aggressive and earlier recurrence [88].
- Delayed graft function, ischemia–reperfusion injury, and post-transplant infection can activate the complement system, potentially leading to early recurrence, especially in patients with complement dysregulation [88].
- Immunological Factors: Genetic or acquired alternative complement pathway abnormalities (e.g., factor H or I mutations, C3NeFs) are linked to a high risk of C3G recurrence after transplant, but the specific predictive value of genetic versus acquired causes is not fully defined per KDIGO guidelines [53,84,89,90].
- Donor Factors: While some reports suggest increased C3G recurrence with living donors, this is not consistently replicated [84].

Immunological and Pathological Mechanisms: MPGN/C3G recurrence post-transplant stems from unaddressed complement dysregulation, leading to uncontrolled alternative complement pathway (ACP) activation. This dysregulation can be inherited (e.g., *CFHR5*, *CFHR3-1* mutations) or acquired (e.g., autoantibodies like C3 nephritic factor, anti-CFH antibodies, or monoclonal immunoglobulins inhibiting complement regulatory proteins or acting as C3NeF). Ischemia–reperfusion injury and post-transplant infections can also trigger ACP. Histologically, recurrence shows mesangial hypercellularity, endocapillary proliferation, MPGN patterns, or crescentic GN with dominant C3 deposition on immunofluorescence (intensity ≥ 2+) [88,91]. Electron microscopy reveals characteristic dense deposits (in DDD) or electron-dense deposits in subendothelial, mesangial, or subepithelial spaces.

##### Treatment of IC-MPGN Recurrence Post-Kidney Transplantation

Underlying causes (e.g., hepatitis C, infections, dysproteinemia, malignancy) of IC-MPGN should be ruled out and treated. Idiopathic cases require individualized therapy due to limited data and varied outcomes.

Glucocorticoids are initial immunosuppressants. Alternatives like mycophenolate mofetil, rituximab [92,93], or cyclophosphamide [94] are used if there are contraindications, intolerance, or inadequate response. KDOQI and KDIGO guidelines support this, emphasizing individualized therapy based on patient risk and comorbidities [92,95]. There is emerging data with pegcetacoplan in IC-MPGN and C3G, as stated below.

##### Treatment of C3G Recurrence Post-Kidney Transplantation

RAAS blockers and lipid-lowering agents are used as supportive treatment. Standard immunosuppressants often fail to prevent C3G recurrence and progression [88]. For C3G recurrence, targeting complement inhibition is crucial. Eculizumab (C5 antibody) for recurring C3G and DDD yields variable results, with some patients showing improved graft function and reduced proteinuria, and others showing no response. This is likely due to diverse complement pathway dysregulation. It is most effective in terminal pathway activation, but consistent patient response is not guaranteed [88,89,90,91,96].

Iptacopan, a factor B inhibitor, shows promise for post-transplant C3G recurrence, improving clinical, lab, and histological markers [97]. Early studies of pegcetacoplan, a C3 inhibitor for C3G (including post-transplant recurrent DDD), show promise in pediatric patients with native kidney C3G [98,99]. These studies indicate it can reduce proteinuria, raise C3 levels, and stabilize or improve kidney function with good short-term safety. The Phase 2 NOBLE study (NCT04572854) demonstrated that pegcetacoplan reduced C3c staining and proteinuria, stabilized eGFR, and improved complement biomarkers in kidney transplant recipients with recurrent C3G or IC-MPGN, compared to the standard of care [100]. VALIANT study (NCT05067127) showed positive results in IC-MPGN/C3G, reducing proteinuria and C3c staining, and stabilizing kidney function. The KDOQI commentary on the 2021 KDIGO guideline emphasizes the need for clinical trial enrollment for C3G due to limited evidence and the potential for personalized complement-targeted treatments.

Screening and monitoring of MPGN/C3G recurrence: Recurrent MPGN or C3G involves close clinical and lab monitoring, including serial creatinine/eGFR, regular proteinuria/hematuria checks, and periodic complement level assessments (C3, C4) with monoclonal protein screening. Protocol (surveillance) kidney biopsies are highly recommended, especially in the first 1–2 years, as early recurrence is often subclinical [101,102]. Prompt biopsies are crucial for unexplained graft dysfunction, new or worsening proteinuria, or hematuria. Complement biomarker profiling and genetic testing are considered in select cases to guide prognosis and therapy. Despite these guidelines, no standardized protocol exists; monitoring is individualized based on risk factors, disease subtype, and evolving evidence [37,88,103].

#### 2.2.5. Anti-Glomerular Basement Membrane (Anti-GBM) Disease

Prevalence and Time to Recurrence: Anti-GBM disease recurrence post-transplant is rare (1.9–4%), attributed to its monophasic nature and sustained immunosuppression [104]. One study found only one recurrence in 53 patients, occurring 5 years post-transplant [104].

Graft Outcome: Anti-GBM disease recurrence is rare but strongly linked to graft loss, often causing rapid, irreversible failure within weeks to months. Due to this low recurrence, long-term patient and graft survival post-transplant are excellent, comparable to other immune-mediated diseases.

Risk Factors:

- Immunological Factors: Circulating anti-GBM antibodies at transplant are a key risk factor for graft loss due to recurrence [104]. KDIGO guidelines advise delaying transplantation until antibodies are undetectable for at least 6 months [10,95].
- Immunosuppression: Cessation or reduction of immunosuppressive drugs is a significant risk factor for reactivation [104]. Patients on low-dose or no immunosuppression are at higher risk.
- Other Factors: Other potential risk factors, such as the pre-transplant disease course, time from initial disease to transplantation, or donor type, have not been shown to predict recurrence [10,85].

Atypical anti-GBM, characterized by linear GBM staining for monotypic or polytypic Ig without a diffuse crescentic pattern, is common post-transplant. A case series of 6 patients found that recurrence averaged 3.8 months post-transplant. All had monotypic Ig staining, negative serum anti-GBM antibodies, and negative serum monoclonal disease and experienced benign courses with no graft loss. Most were on maintenance immunosuppression with good outcomes; 3 received plasma cell therapies due to identifiable clones [105].

De novo anti-GBM disease, a rare complication (2–3% of cases) of post-transplant Alport disease, stems from an alloimmune response to neoantigens within the allograft. Although maintenance immunosuppression can mitigate glomerular involvement, the presence of this disease leads to rapid graft loss. Subsequent transplants may result in more aggressive disease [95].

Immunological and pathological mechanisms:

Recurrence of anti-GBM disease post-kidney transplant is caused by the re-emergence of anti-GBM antibodies that target the non-collagenous domain of the α3 chain of type IV collagen within the glomerular basement membrane. The precise immunological mechanisms driving recurrence are not fully understood, but potential triggers include the cessation or reduction of immunosuppression, which can reactivate anti-GBM antibodies. Additionally, cellular rejection may expose previously hidden epitopes, thereby inducing the formation of antibodies against them. The existence of antibody-negative cases highlights the limitations of relying solely on serological monitoring [106,107].

The highest risk of recurrence is observed in patients with detectable circulating anti-GBM antibodies at the time of transplant. While recurrence is uncommon when transplantation occurs during serological quiescence, if it does happen, it leads to rapid and severe allograft injury, often manifesting as crescentic glomerulonephritis. Immunopathology typically reveals linear IgG deposition along the GBM, complement activation, and inflammatory cell recruitment.

Treatment of recurrent anti-GBM disease:

Recurrent post-transplant anti-GBM disease needs immediate treatment: high-dose corticosteroids (e.g., IV methylprednisolone 500–1000 mg daily for 3 days, then oral prednisone 1 mg/kg/day, tapered clinically), daily/QOD plasma exchange until anti-GBM antibodies are undetectable, and cyclophosphamide (oral or IV, adjusted by kidney function and cytopenias). Refractory cases may use rituximab or immunoadsorption, as per KDIGO guidelines and case series [16,78,95,106]. Once antibodies are undetectable, maintenance immunosuppression beyond standard post-transplant care is usually unnecessary.

Screening and monitoring of anti-GBM recurrence: Post-transplant, it is recommended to screen for microhematuria and graft dysfunction through urinalysis, initially at baseline, then quarterly for the first year, and subsequently annually. If recurrence is suspected, an allograft biopsy is recommended for confirmation and to rule out other potential causes [16,95,102].

#### 2.2.6. Lupus Nephritis (LN)

Prevalence and Time to Recurrence: Recurrent lupus nephritis (RLN) after kidney transplantation varies widely, from 2.44% in registry analyses [108] to 30–54% in single-center reports [109]. This is due to differing biopsy practices (for-cause vs. protocol), follow-up duration, population, and immunosuppressive regimens [108,110,111]. RLN can occur anytime, from one week to 16 years post-transplant, though most events occur within 10 years. Patient demographics, ANA, dsDNA, and complement activity are poor relapse markers; new-onset proteinuria and hematuria are the clinical indicators of biopsy.

Graft Outcome: Severe RLN, though uncommon, raises graft failure risk significantly; one study showed 93% failure with RLN versus 19% in controls, and a fourfold greater risk than without recurrence [108]. However, RLN accounts for only 7% of overall graft failure, compared to 43% for rejection. Patient survival rates are similar for RLN and rejection patients, and not notably lower than controls without recurrence [108].

Risk Factors:

- Recipient Factors: Non-Hispanic Black race, female gender, age <33, dialysis pre-transplant [108], and the presence of antiphospholipid antibodies [109,112]. High SLEDAI or serologic activity at transplant may also increase risk, though the American College of Rheumatology (ACR) states that serologic activity alone should not preclude transplantation [111].
- Donor Factors: Recipients of RLN commonly received a deceased-donor kidney allograft. High levels of HLA-A and HLA-B locus mismatch in deceased-donor transplants, and a high frequency of zero-haplotype match with living donors, are also associated with increased risk [108]. Some studies suggest a higher recurrence rate with living donor kidneys, though this finding is not consistent across all cohorts [109].
- Immunosuppression: Mycophenolate reduces RLN incidence [108]. Though maintenance azathioprine hinted at a non-significant trend towards higher risk, the type of induction or maintenance immunosuppression was not a strong independent predictor in multivariate analyses [108]. Lack of induction therapy correlates with increased recurrence and poorer outcomes in some studies [113,114].

Immunological and Pathological Mechanisms:

Recurrent lupus nephritis (RLN) after kidney transplant is caused by the reactivation of systemic autoimmunity, leading to immune complex deposition in the allograft, driven by autoantibodies (such as anti-dsDNA), hypocomplementemia, and immune complex-mediated glomerular injury. Post-transplant immunosuppression usually keeps recurrence low and mild. However, recurrence can happen in patients with active serology at transplant (elevated dsDNA and low complement) or inadequate immunosuppression [114,115]. KDIGO [110] and ACR Guidelines [111] recommend regular monitoring due to potential subclinical recurrence and the risk of extrarenal SLE flares. Non-immune complex mechanisms, such as thrombotic microangiopathy and atypical pauci-immune proliferative GN and FSGS [116], also contribute. Overall, recurrent LN is influenced by persistent autoimmunity and immunosuppression, with most recurrences subclinical and rarely causing graft loss.

##### Treatment of Recurrent LN (RLN) Post-Kidney Transplantation

RLN post-transplant is managed by adjusting or escalating baseline immunosuppression. This typically involves a calcineurin inhibitor (CNI), mycophenolate, and pulse glucocorticoids, tailored to the lupus nephritis (LN) class, severity, and prior treatment response. For severe or refractory cases, treatment may escalate to triple therapy: glucocorticoids, mycophenolic acid (MPA), and either belimumab or a CNI, or cyclophosphamide and belimumab. If MPA is ineffective, cyclophosphamide may be considered. Rituximab may be added for refractory cases. Intensified immunosuppression generally continues for 3–5 years after a complete renal response before tapering. It is important to note that most of the treatment data for LN is derived from native kidney disease [110,111,117].

Before intensifying immunosuppression, guidelines from the ACR [111] and KDIGO [110] recommend a repeat kidney biopsy. This is crucial to differentiate active lupus nephritis (LN) from chronic allograft injury or other causes of kidney dysfunction. Additionally, supportive therapies like RAAS inhibitors and hydroxychloroquine should be continued as appropriate, unless there are contraindications.

Screening and monitoring of recurrent lupus nephritis: Monitoring for RLN requires regular clinical and laboratory monitoring, including renal function, urinalysis, UPCR, and serologic markers such as dsDNA and complement (with clinical correlation). ACR [111] and KDIGO [110] emphasize follow-up due to the risk of recurrence, although this risk is low, and flares are often mild. Persistent renal abnormalities warrant biopsy to distinguish lupus nephritis from allograft injury. Protocol biopsies are not routinely recommended, although they can detect subclinical recurrences; most centers prefer indication biopsies.

#### 2.2.7. ANCA-Associated Vasculitis (AAV)

Prevalence and Time to Recurrence: The recurrence of AAV in kidney allografts is estimated at 0.1 per patient per year, with both early and late recurrences. Early recurrence (within weeks) causes primary graft non-function and extrarenal symptoms; high seropositivity was linked to recurrence despite clinical remission. Late recurrence, years post-transplantation, had an insidious course, making diagnosis challenging despite multiple kidney biopsies, and progressed to ESRD [118].

Graft Outcome:

AAV recurrence post-transplant is rare (<10%), typically occurring beyond the first post-transplant year. Risk factors (pre-transplant course, ANCA subtype/titer, donor type) are not well-established predictors of recurrence. Though rare, recurrence threatens graft survival and necessitates prompt management. Graft loss more often stems from death with a functioning graft, commonly due to immunosuppression-related infection and malignancy, and not disease recurrence [10,119,120,121].

Risk Factors:

- Immunological Factors: Persistent post-transplant ANCA modestly elevates risk but is not a strong relapse predictor without confirmation of active disease and does not warrant immunosuppression escalation. ANCA positivity should not delay transplant or impact relapse rates. Close monitoring is recommended for patients with persistently positive titers [95,122].
- Donor Factors: Donor factors (type, age, sex, HLA matching) do not significantly influence AAV recurrence risk after kidney transplantation [10,122,123].
- Recipient Factors: Recipient age, disease duration, and comorbidities do not significantly influence AAV recurrence risk post-transplant [122].
- Immunosuppression: Modern regimens (calcineurin inhibitors, mycophenolate mofetil, corticosteroids) reduce relapse rates [121]; standard protocols are recommended, and prophylactic immunosuppression with RTX is not recommended.
- Timing of Transplant: Receiving the transplant while ANCA vasculitis is still active carries a high risk of recurrence and mortality, especially PR3 positivity [53]. Persistent ANCA should not delay transplantation. Guidelines recommend delaying kidney transplantation until patients are in complete remission for at least 6 months, or ideally 12 months, despite persistent ANCA antibodies [122].

Immunological and Pathological Mechanisms: AAV recurrence after kidney transplant results from reactivated autoimmunity against neutrophil antigens (PR3, MPO), causing pauci-immune necrotizing and crescentic glomerulonephritis in the allograft, sometimes accompanied by arteritis or granulomatous vasculitis. Persistent ANCA drives recurrence by activating neutrophils/monocytes, leading to endothelial injury and small-vessel vasculitis. Theories for recurrence include incomplete T-helper cell inhibition by transplant immunosuppressants and epitope spreading beyond PR3/MPO [10,120].

##### Histology

Clinical presentations often include rising serum creatinine, hematuria, and proteinuria and rising ANCA titers. Histopathologically, recurrence is characterized by pauci-immune necrotizing and crescentic glomerulonephritis, and may also present as acute arteritis or, rarely, granulomatous vasculitis, causing ureteral stenosis or obstructive uropathy. Biopsy is essential to confirm active disease, distinguish recurrence from chronic injury or rejection, and guide therapy [10,122].

##### Treatment of AAV Recurrence Post-Kidney Transplantation

Rapid reinduction of remission is critical. High-dose glucocorticoids are the cornerstone of initial therapy due to the risk of rapid allograft loss if vasculitis is uncontrolled. Rituximab is preferred for relapsing AAV post-transplant due to its efficacy and favorable safety profile compared to cyclophosphamide, especially with prior cyclophosphamide exposure [120,124,125]. KDIGO 2024 guidelines recommend rituximab first-line for relapsing/refractory disease, with cyclophosphamide as an alternative [95,122]. Plasma exchange may be added for severe/refractory, life-threatening cases [122,126].

Managing refractory disease may involve switching rituximab/cyclophosphamide, increasing glucocorticoids, or adding plasma exchange, tailored to patient factors like prior drug exposure, comorbidities, and infection risk. Evidence in transplant patients is limited, relying on native kidney disease data and small case series; further research is needed [122].

Screening and monitoring of AAV recurrence: Post-kidney transplant, AAV recurrence requires close clinical surveillance, including structured assessments, inflammatory markers, and kidney function tests (eGFR, creatinine, proteinuria, hematuria). Recurrence risk is low, but vigilant monitoring is crucial, as relapses can occur later and present as pauci-immune GN or extrarenal relapses. While routine ANCA titer monitoring can be performed, changes are only modestly predictive of relapse and should not be the sole guide. Persistent or rising ANCA, especially in PR3-ANCA patients, may necessitate closer observation, but ANCA positivity at transplant does not reliably predict recurrence. There is no consensus on monitoring frequency, but continuous vigilance for clinical and lab evidence of recurrence is recommended. If suspected, prompt evaluation (including kidney biopsy and consideration of reinduction with rituximab) is advised [95,122].

## 3. Conclusions

Recurrent GN post-kidney transplant remains a challenge in transplant nephrology. Transplantation improves ESRD patient survival and quality of life but does not cure the underlying systemic disease that initially caused kidney failure. Therefore, the allograft is susceptible to GN recurrence, affecting long-term graft survival.

Post-transplant GN recurrence varies greatly by subtype, from rare in anti-GBM to almost universal in DDD, emphasizing the need for accurate pre-transplant diagnosis and risk stratification. FSGS and MPGN/C3G carry high recurrence and early graft loss risks. IgAN frequently recurs but has better long-term graft outcomes. Lupus nephritis and AAV have lower recurrence rates in allografts; graft loss is often due to immunosuppression complications (infection, malignancy) rather than direct disease recurrence. The risk of recurrence of 3–15% is an underestimate. A total of 45% of allografts fail within 5 years of recurrence [3,6,7,8], and recurrent GN is the third leading cause of graft loss at 10 years. Recurrent GN diagnosis hinges on clinical suspicion (proteinuria, hematuria, declining graft function) and renal allograft biopsy (light microscopy, immunofluorescence, electron microscopy). While “for-cause” biopsies are common, “protocol” biopsies reveal more subclinical recurrences and a higher true incidence. Monitoring strategies are GN subtype-specific, using serological markers and varied clinical/lab assessments.

Recurrence mechanisms are diverse, often involving persistent circulating factors in FSGS, autoantibodies (PLA2R in MN, anti-dsDNA in RLN, ANCA in AAV), or complement dysregulation in C3G. Genetic predispositions are key in IgAN and C3G. Recurrence risk factors include recipient characteristics (younger age, rapid progression to ESRD, prior recurrence), immunological markers (antibody titers, complement levels, presence of monoclonal gammopathy), and aspects of transplant management (e.g., immunosuppressive regimens, type of donor).

This narrative review has several limitations, including potential selection bias due to non-systematic study inclusion and possible omission of relevant studies. A formal risk-of-bias assessment or meta-analysis was not performed, so findings should be interpreted with caution. The evidence base is heterogeneous across diseases and study designs, with variability in diagnostic criteria and outcome definitions. Most data are derived from retrospective cohorts and transplant registries, which are subject to indication and survivorship biases. Across subtypes, outcome reporting is inconsistent, and response criteria are not standardized in the transplant setting. Randomized controlled trials specifically in kidney transplant recipients are scarce, and the generalizability from native kidney disease studies is uncertain. Future efforts should focus on developing and validating predictive biomarkers for recurrence risk stratification pre- and post-transplant by disease subtype. It is also important to establish standardized diagnostic and response criteria tailored to the transplant context, including biopsy and non-invasive markers. Conducting multicenter prospective cohorts and pragmatic trials to evaluate therapies and monitoring strategies in transplant recipients and creating shared registries with harmonized data elements to enable comparative effectiveness analyses across centers would also be beneficial.

In conclusion, the recurrence of GN post-kidney transplantation is a complex and significant determinant of long-term graft success. The differential impact of recurrence across GN subtypes necessitates highly individualized patient management, from pre-transplant counseling and risk assessment to tailored immunosuppression and vigilant post-transplant monitoring. Continued research into the precise immunopathogenesis of each GN type, the identification of reliable predictive biomarkers, and the development of targeted therapeutic interventions are paramount to improving outcomes for kidney transplant recipients facing this persistent challenge.

**Figure 1:**
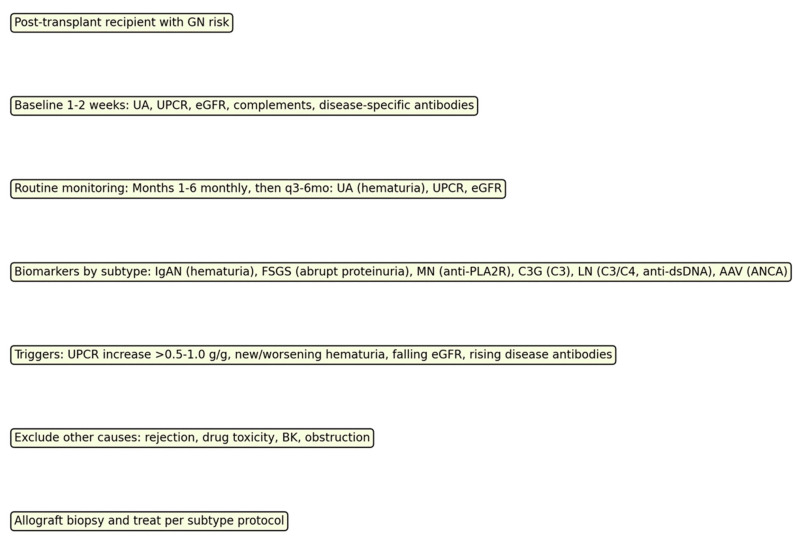
Monitoring algorithm for post-transplant surveillance and biopsy thresholds by subtype.

**Table 1:** Incidence and time to recurrence by GN subtypes.

Subtype	Reported Recurrence Rate	Time to Recurrence	Impact on Graft Survival
Overall recurrent GN	3–15% (likely an underestimate).	Increases with time; the 2nd most common biopsy finding at 10 years.	45% of grafts fail within 5 years of recurrence [3,6,7,8]; 3rd leading cause of graft loss at 10 years.
IgA nephropathy (IgAN)	10–30% (indication biopsy); 25–53% (protocol biopsy) [9,10,11,12]; up to 51% at 5 years.	Median ~59 months (range 16–90 months); clinical recurrence often after 5 years.	Graft loss in up to 40%; ~42% 5–year graft failure; accounts for 60% of graft failures in this group.
FSGS	Recurrence 40–60%; up to 80% in high-risk cohorts [37].	Recurrence often occurs within days to months (median 1.5 months) post-transplant, mostly within the first two years [36,37].	Recurrent FSGS significantly reduces 5-year graft survival to 52%, compared to 83% in non-recurrent cases, with most graft losses occurring within two years of recurrence. De novo FSGS offers better long-term graft survival (60%) than recurrent FSGS (33.3%), with slower progression [38].
Membranous nephropathy	10–45%, 31% incidence at 10 years.	Median 6.3 years, longer than other GN. Early recurrence (6–12 months) suggests pre-existing antibodies; late onset (~5 years) implies new antibody production.	Better in recurrent membranous nephropathy compared to de novo (11.7/100 person-years vs. 3.7/100 person-years [68]).
MPGN/C3 glomerulopathy (C3G, DDD)	C3G and monoclonal IC-MPGN have 20–50% higher recurrence rates, with C3G exceeding 60–70%. DDD almost always recurs (near 100%).	C3G often recurs early post-transplant [37,82,83], sometimes within 9 days (median 1.1–28 months) [84].	Recurrent MPGN and C3G lead to poor graft outcomes, with C3G recurrence causing graft failure in 11–77% (mostly >50%) [82,83,84,85,86]. Graft loss is more frequent and rapid in DDD than in C3 glomerulonephritis [84].
Anti-GBM disease	Rare 1.9–4% [104].	Early recurrence is related to circulating antibodies at transplant.	Post-transplant GN recurrence is rare, but links to graft loss. Overall, patient and graft survival are excellent, and atypical anti-GBM outcomes are favorable.
Lupus nephritis (LN)	2.44% in registry analyses [108] (high as 30–54% in single-center cohorts based on biopsy patterns [109]).	1 week to several years, most within 10 years.	Rarely causes graft loss (7%); severe LN is uncommon but has higher graft loss. One study revealed 93% failure with RLN versus 19% in controls, and a fourfold greater risk than without recurrence [108].
ANCA-associated vasculitis	Rare, 0.1 per patient per year, with both early and late recurrences.	Early recurrence (weeks) leads to primary graft non-function and extrarenal symptoms. Late recurrence (years) is insidious, hard to diagnose, and progresses to ESRD despite biopsies [118].	Graft loss often results from death with a functioning graft, frequently due to immunosuppression-related infection and malignancy, not disease recurrence [10,119,120,121].

**Table 2:** Risk factors for recurrent GN after kidney transplantation.

Subtype	Risk Factors
IgA nephropathy	Risk factors for post-transplant IgAN recurrence include younger age at diagnosis/transplant [9,15], rapid ESRD progression, prior transplant, pre-transplant hemodialysis [15], steroid withdrawal/sparing, mTOR inhibitor use [15,17], elevated Gd-IgA1 and anti-Gd-IgA1 [18], low HLA-DR mismatch [15], living donor status, crescentic disease, rapid pre-transplant eGFR decline [20], and presence of pre/post-transplant DSAs [9].
FSGS	Risk factors for recurrent FSGS include younger age, rapid progression to ESRD [39,40], white race, pre-transplant antinephrin antibodies [41,42], prior recurrent FSGS (80% risk), and nephrotic syndrome at native disease onset [39,40].
Membranous nephropathy	Risk factors include elevated anti-PLA2R pre-transplant [7,37,71,72,73], female sex, younger age [5], recipient HLA-A3, living-related transplants [68], high pre-transplant proteinuria [72], faster ESRD progression [71], and donor HLA-D and PLA2R1 risk alleles [74,75].
MPGN/C3G	Pre-transplant low complement, monoclonal gammopathy [1,53,82], C3 nephritic factor, and genetic abnormalities (CFH, CFI, MCP) [53,84,89,90] are risk factors. Other factors include young age, crescents in original biopsy, low BMI [10,85], shorter dialysis duration before kidney transplantation [87], and prior graft loss from MPGN [10].
Anti-GBM disease	Pre-transplant circulating antibodies [104] and post-transplant cessation or reduction in immunosuppression [104] may lead to recurrence. Factors like pre-transplant disease course, time to transplant, or donor type do not predict recurrence [10,85].
Lupus nephritis	Risk factors include African American or Hispanic ethnicity, younger age, antiphospholipid antibodies [109,112], pre-transplant dialysis [108], HLA-A and HLA-B locus mismatch (deceased-donor), high frequency of zero-haplotype match (living donors) [108], and lack of induction therapy [113,114]. MMF induction reduces recurrence [108].
ANCA-associated vasculitis	Increased post-transplant ANCA titers do not strongly predict relapses or warrant immunosuppression escalation. CNI, MMF, and steroids reduce relapse risk [121]. Donor factors [10,122,123] (type, age, sex, HLA matching), recipient age, disease duration, and comorbidities do not significantly influence AAV recurrence risk [122]. Active ANCA vasculitis at transplant, especially PR3 positivity, carries a high risk of recurrence and mortality. Persistent ANCA should not delay transplantation; guidelines recommend delaying until complete remission for 6–12 months despite persistent ANCA antibodies [122].

**Table 3:** Management and prognosis of recurrent GN after kidney transplantation.

Subtype	Management Strategies	Prognosis
IgA nephropathy	Supportive therapy includes RAAS blockade [10] and maintaining corticosteroids, avoiding early steroid withdrawal. For active/severe recurrence, IV MP (500 mg daily or 3 days at months 1, 3, 5) combined with oral prednisone (0.5 mg/kg every other day for six months) may be considered [23]. Experimental treatments for severe/treatment-resistant cases (especially with endocapillary proliferation or crescentic lesions) include cyclophosphamide [24,25,26], rituximab [27,28], and eculizumab [29]. Tonsillectomy is reported to be beneficial in Japan [30]. Novel IgAN therapies (BAFF–APRIL inhibitors) are under investigation [32,33].	Frequent recurrence but relatively favorable compared with other GN [5,9,10,13,14]; graft loss in up to 40%.
FSGS	First-line treatment: plasmapheresis ± rituximab [39] (early RTX benefit), high-dose steroids, intensified calcineurin inhibitors (cyclosporine/tacrolimus). For PLEX or RTX unresponsive cases: ACTH gel [61,62], LDL pheresis [63,64,65], abatacept, and ofatumumab [66,67].	Early/treatment-resistant recurrence of FSGS has a poor prognosis, with up to 50% graft loss. De novo FSGS offers better graft survival (60%) than recurrent FSGS (33.3%) [38].
Membranous Nephropathy	Adhere to existing transplant immunosuppressants and antiproteinuric agents. Rituximab is preferred first-line immunosuppression [71,72,78], while obinutuzumab has been used for rituximab-resistant recurrent MN in case reports and series [79,80,81].	Favorable outcome with graft survival.
MPGN/C3G	For IC-MPGN, glucocorticoids are the initial immunosuppressants. Mycophenolate mofetil, rituximab [92,93], or cyclophosphamide [94] are alternatives if glucocorticoids are contraindicated, not tolerated, or ineffective; complement inhibition (eculizumab, ravulizumab) in C3G/DDD; Iptacopan [97] and pegcetacoplan in C3G have shown promise [98,99]; supportive therapy with RAAS blockade; treatment of underlying disease.	High recurrence, poor graft survival, especially in DDD.
Anti-GBM disease	High-dose corticosteroids, daily PLEX (until antibody titers negative), and oral cyclophosphamide are primary treatments. Rituximab may benefit refractory cases.	Recurrence is rare but associated with graft loss.
Lupus nephritis	Standard immunosuppression (steroids, MMF, calcineurin inhibitors) should be maintained and escalated as needed. Post-transplant, add hydroxychloroquine. For refractory disease, consider cyclophosphamide, belimumab, or rituximab.	Generally indolent; rarely causes graft failure; outcomes favorable with adequate immunosuppression.
ANCA-associated vasculitis	High-dose corticosteroids; rituximab preferred over cyclophosphamide for relapsing disease. PLEX for life-threatening cases.	Good prognosis with early recognition and treatment.

**Table 4:** Screening and monitoring for the risk of recurrence.

Disease	Key Monitoring Parameters and Tests	Recommended Frequency	Biopsy Guidance and Key Notes
IgA Nephropathy (IgAN)	Urine for microhematuria, proteinuria, creatinine, and eGFR	Monthly for the 1st month, then quarterly for the 1st year, then annually.	Kidney biopsy is indicated for new/worsening disease or graft dysfunction [10,16]. Investigational biomarkers (e.g., Gd-IgA1) are not standard.
Recurrent FSGS (rFSGS)	Proteinuria (spot urine UPCR/UACR), creatinine, eGFR	High-Risk Patients: Daily for 1 week, weekly for 4 weeks, quarterly for 1 year, then annually.	Recurrence is confirmed by kidney biopsy [10,16]. Pre-transplant genetic testing is vital to exclude genetic forms. Investigational biomarkers include suPAR, CLC-1, AT1R-Ab, anti-CD40, IL-13, and antinephrin antibodies.
Recurrent Membranous Nephropathy (MN)	Proteinuria (spot urine UPCR/UACR), creatinine, eGFR, anti-PLA2R antibodies (if PLA2R-positive MN)	Proteinuria: Monthly for 6–12 months. Anti-PLA2R: Monthly to quarterly, per pre-transplant levels.	Kidney biopsy indicated for proteinuria >1 g/day or rising anti-PLA2R (0.3–1.0 g/day) [71,78]. For non-PLA2R MN, biopsy if proteinuria >1 g/day [78].
MPGN/C3G	Proteinuria (spot urine UPCR/UACR), eGFR/creatinine, hematuria, Complement levels (C3, C4) with monoclonal protein screening	No single standard protocol exists; monitoring is regular but individualized based on the patient’s case.	First 1–2 years post-transplant, surveillance biopsies are highly recommended due to potential subclinical recurrence [101,102]. Prompt biopsies are vital for unexplained graft dysfunction, new/worsening proteinuria, or hematuria. Complement biomarker profiling and genetic testing are considered in specific cases for prognosis and therapy. Despite guidelines, no standardized protocol exists; monitoring is individualized based on risk factors, disease subtype, and evolving evidence [37,88,103].
Anti-GBM Disease	Urinalysis (for microhematuria) and graft function (eGFR, creatinine)	Quarterly for the 1st year, then annually.	Kidney biopsy is recommended to confirm a suspected recurrence [16,95,102].
Lupus Nephritis (RLN)	Kidney function (eGFR/creatinine) Urinalysis/UPCR Inflammatory markers (CRP, ESR) Serologic markers (dsDNA, complement)	Regular, long-term monitoring is required, but a specific schedule is not standardized.	ACR [111] and KDIGO [110] highlight the importance of follow-up for recurrence risk, despite it being low and flares usually mild. Persistent renal abnormalities require biopsy to differentiate lupus nephritis from allograft injury. Protocol biopsies are not standard; indication biopsies are preferred.
ANCA-Associated Vasculitis (AAV)	Kidney function (eGFR/creatinine) and urinalysis (for hematuria and proteinuria), inflammatory markers (CRP and ESR), ANCA titers	No consensus on frequency; requires continuous clinical vigilance.	Kidney Biopsy: Advised for prompt evaluation if recurrence is suspected [95,122]. Notes: ANCA levels alone are not reliable predictors of relapse.

## Data Availability

No new data were created or analyzed in this study as this is a narrative review. All the references have been listed. Data sharing is not applicable to this article.

## Abbreviations

AAV	ANCA-associated vasculitis
DDD	dense deposit disease
ESRD	end-stage kidney disease
FSGS	focal segmental glomerulosclerosis
GBM	glomerular basement membrane
GN	glomerulonephritis/glomerular diseases
KDIGO	Kidney Disease: Improving Global Outcomes
LN	lupus nephritis
MMF	mycophenolate mofetil
MN	membranous nephropathy
MPGN	membranoproliferative glomerulonephritis
RAAS	renin–angiotensin–aldosterone system
